# Current Practices of Prone Positioning in ARDS Reported by European ICU Clinicians (CUPIDO)—A Cross-Sectional Questionnaire-Based Survey

**DOI:** 10.3390/jcm15208082

**Published:** 2026-10-18

**Authors:** Fabian Gleibs, Markus Haar, Denise Battaglini, Silvia De Rosa, Sergio Lassola, Aneta Volfová, Lise Piquilloud, Stefan Kluge, Marcus J. Schultz, Kevin Roedl

**Affiliations:** 1 Department of Intensive Care Medicine, University Medical Centre Hamburg–Eppendorf, 20246 Hamburg, Germany; m.haar@uke.de (M.H.); anetavolfova1@gmail.com (A.V.); s.kluge@uke.de (S.K.); k.roedl@uke.de (K.R.); 2 Department of Surgical Sciences and Integrated Diagnostics, University of Genoa, 16132 Genoa, Italy; battaglini.denise@gmail.com; 3 Department of Anaesthesia and Intensive Care, IRCCS Azienda Ospedaliera Metropolitana, 16132 Genoa, Italy; 4 Centre for Medical Sciences (CISMED)–Interdepartmental Centre for Medical Sciences, University of Trento, 38122 Trento, Italy; silvia.derosa@unitn.it (S.D.R.); sergio.lassola@apss.tn.it (S.L.); 5 Department of Anaesthesia and Intensive Care, Azienda Sanitaria Universitaria Integrata del Trentino (ASUIT), Santa Chiara Ospedale, 38122 Trento, Italy; 6 Adult Intensive Care Unit, University Hospital, University of Lausanne, 1011 Lausanne, Switzerland; lise.piquilloud@chuv.ch; 7 Department of Anaesthesia, General Intensive Care and Pain Management, Division of Cardiothoracic and Vascular Anaesthesia & Critical Care Medicine, Medical University Wien, 1090 Vienna, Austria; marcus.j.schultz@gmail.com; 8 Department of Anaesthesiology, Rescue and Pain Medicine, Cantonal Hospital St. Gallen, HOCH Health Ostschweiz, 9007 St. Gallen, Switzerland; 9 Nuffield Department of Medicine, University of Oxford, Oxford OX3 7BN, UK; 10 MahidolOxford Tropical Medicine Research Unit (MORU), Mahidol University, Bangkok 10400, Thailand

**Keywords:** acute respiratory distress syndrome, prone positioning, respiratory medicine, critical care, intensive care medicine

## Abstract

**Background**: Prone positioning improves survival in patients with moderate to severe acute respiratory distress syndrome (ARDS), yet its implementation in routine clinical practice remains poorly characterized. We conducted a survey to assess contemporary prone positioning practices and their heterogeneity in the management of ARDS across European intensive care units (ICUs). **Methods**: A cross-sectional, questionnaire-based survey was conducted among clinicians involved in ARDS management between March and August 2025. The survey was developed by a panel of experts in intensive care medicine and underwent testing for face and content validity prior to dissemination. The questionnaire was distributed through European networks, including the European Society of Intensive Care Medicine (ESICM), the PROtective VEntilation Network (PROVE Network), the European Society of Anaesthesiology and Intensive Care (ESAIC), and national societies. **Results**: Out of 592 eligible responses, 451 responses were complete and from European clinicians (66.1% male), with the largest contributions being from Italy (28.6%), Germany (18.4%) and Austria (11.5%). Of these, 446 respondents (98.9%) reported performing prone positioning. Initiation thresholds for prone positioning varied, most commonly a ratio of arterial partial pressure of oxygen to fraction of inspired oxygen (PaO_2_/FiO_2_) ≤ 150 mmHg (55.4%), followed by ≤100 mmHg (26.9%), ≤200 mmHg (11.4%), and ≤300 mmHg (1.8%). Full prone positioning was reported as *always* or *often* used by 38.6% and 37.7% of participants, whereas semi-prone positioning (7.0% and 21.1%) or lateral positioning (2.5% and 14.8%) was used less often. The duration of prone positioning varied: 47.8% reported 16 h, 17.0% 12 h and 7.8% 8 h per session. **Conclusions**: Among self-selected clinicians working in European ICUs, prone positioning was commonly reported, though reported practice varied with respect to initiation thresholds, prone positioning strategy and duration. These findings identify specific areas where practice diverges and where current guidance and evidence remain incomplete.

## 1. Background

Prone positioning is one of the few interventions shown to improve survival in patients with acute respiratory distress syndrome (ARDS). Early randomized trials failed to demonstrate a clear mortality benefit [1,2,3,4], but the landmark ‘Prone Positioning in Severe Acute Respiratory Distress Syndrome’ (PROSEVA) trial established a significant survival advantage when prone positioning was applied early, completely, for prolonged sessions, and in patients with moderate to severe hypoxemia [5].

The external validity of the pivotal evidence warrants careful consideration. PROSEVA applied strict inclusion criteria based on PaO_2_/FiO_2_ (partial pressure of oxygen in arterial blood/fraction of inspired oxygen) thresholds and used a highly standardized protocol with predefined session durations and experienced centers. Since its publication, several modifications of prone positioning strategies have emerged, including variations in initiation thresholds [6], session durations [3,7,8], sedation and neuromuscular blockade practices [9,10], and discontinuation criteria [11]. Moreover, prone positioning has been extended to non-intubated patients [12,13,14], thereby moving beyond the original evidence base established in invasively ventilated patients with severe ARDS. The coronavirus disease 2019 (COVID-19) pandemic accelerated the adoption of prone positioning, with use reported in nearly half of Swedish intensive care unit (ICU) patients and almost two thirds of invasively ventilated Italian patients [15,16]. This increased adoption was followed by a substantial decline in the post-pandemic era [17].

Despite these implementation challenges, it remains unclear which patients are currently selected for prone positioning, how consistently evidence-based protocols are applied, and to what extent practices differ across institutions and countries. In parallel, major professional societies such as the American Thoracic Society (ATS) and the European Society of Intensive Care Medicine (ESICM) have issued recommendations that differ in aspects such as the strength of endorsement, timing, and duration of prone positioning [18,19,20], potentially contributing to further heterogeneity in implementation. To better understand contemporary practice across European ICUs, we conducted a European survey assessing current approaches to prone positioning for ARDS. We hypothesized that, given the boundaries of the original evidence and the subsequent diversification of practice, substantial variation exists across European intensive care units.

## 2. Methods

### 2.1. Design

We conducted a cross-sectional, questionnaire-based survey of medical staff involved in the management of ARDS. The data of this study were collected as part of a large survey, named ‘CUrrent Practices of Intensive Care for the Management of Acute Respiratory Distress Syndrome in EurOpe’ (CUPIDO). The complete survey was designed to take no more than 20 min to complete. The survey was prospectively registered at ClinicalTrials.gov (NCT06714201).

Informed consent was obtained on the first page of the online survey. No patient data, names or contact details were collected. Postal codes were used to identify institutions for clustering analysis, with results reported in aggregate. Formal ethical review was not sought, because the survey involved no intervention, patient data, or data attributable to identifiable individuals, in accordance with the German professional code for physicians. Participation was voluntary and no incentives were provided.

This report follows the Strengthening the Reporting of Observational Studies in Epidemiology (STROBE) statement for cross-sectional studies [21] and the Checklist for Reporting Results of Internet E-Surveys (CHERRIES) [22]. Both completed checklists are provided in the Supplementary Material (Methods S1 and S2).

### 2.2. Survey Development and Content

The questionnaire was developed de novo in English by seven senior ICU physicians (>5 years of ICU experience) from five European academic centers (Hamburg, Trento, Genova, Lausanne and Amsterdam). Items were based on the PROSEVA trial [5] and clinical expertise. Each panel member drafted a set of questions, which were reviewed by the full panel in several rounds for relevance, completeness and clarity (face and content validity); disagreements were resolved by consensus. The draft was subsequently pilot tested within the study group by two physicians not involved in its development (one senior and one junior), resulting in minor wording and grammatical changes. No formal quantitative content validity assessment or cognitive interviewing was performed.

The questionnaire was a collaborative effort between the University of Hamburg (Department of Intensive Care Medicine, University Medical Centre Hamburg-Eppendorf), the University of Trento (Centre for Medical Sciences, Santa Chiara Hospital), the University of Genova, the University of Lausanne (Adult Intensive Care Unit), the University of Amsterdam (Department of Intensive Care, Amsterdam UMC) and the PROtective VEntilation (PROVE) Network. Implementation was performed using the online platform SurveyMonkey. Formal endorsement was given by the European Society of Intensive Care Medicine. The detailed questionnaire is provided in the Supplementary Materials (Methods S3).

No question was mandatory, and respondents could skip individual items. Respondents who reported not using prone positioning were automatically directed to the subsequent questionnaire domain. Respondents were included in the analysis if they completed the characteristics section and either reported not using prone positioning or completed the prone positioning section through to its final question. Responses not meeting these criteria were excluded.

### 2.3. Survey Dissemination

The questionnaire was distributed electronically between 7 March 2025 and 31 August 2025. Distribution relied predominantly on national and international professional societies and guideline associations for intensive care medicine, and on professional networks outside of these organizations.

### 2.4. Participants and Eligibility

Eligible participants were healthcare professionals practicing in European ICUs. While the primary target group was medical doctors working in intensive care units, responses from other ICU professionals, such as nursing staff, were also retained.

### 2.5. Outcomes

The main outcomes of this survey were the PaO_2_/FiO_2_ threshold for initiating prone positioning, positioning strategy (full prone, semi-prone or lateral) and session duration, as the three key elements of how prone positioning is applied, as defined in the PROSEVA trial [5], and because we expected them to vary across clinicians.

### 2.6. Statistical Analysis

We restricted this report to respondents who returned a response with a completed characteristics section, to minimize the risk of duplicate entries, and excluded responses from outside of Europe. Categorical variables are reported as counts and percentages. Continuous variables are reported as the median and interquartile range (IQR). Proportions were calculated using the number of respondents per question as the denominator. Denominators varied due to different samples investigated and are reported in the footnotes of each table. Missing responses were not imputed and are reported as such. Institutions were identified by country and the postal code of the respondent’s hospital.

In a post hoc analysis, we compared reported practice with recommendations on prone positioning in the 2023 ESICM guideline [20] and the 2017 ATS guideline [18], focusing on the recommended thresholds for initiation and duration of prone positioning. Next, we assessed country- and institution-level characteristics, including ICU size, academic status, extracorporeal membrane oxygenation availability, and the reported number of healthcare workers involved in repositioning for respondents whose practice was fully consistent with the ESICM guideline and with reported use of awake prone positioning.

Furthermore, we examined independent associations between practice patterns and institutional and clinician characteristics. We constructed three separate multivariable mixed-effects regression models, each with one outcome variable of interest (threshold for initiation, frequency of full prone positioning strategy, and minimum duration of one positioning cycle). Model family was matched to outcome type: ordinal outcomes (threshold for initiation, positioning frequency) were modeled using cumulative link mixed-effects regression, while the continuous outcome (duration) was modeled using a linear mixed model. All models included the same three predictor variables entered jointly: ICU bed capacity (ordinal, ranked 1 to 5 across size categories), clinician age (ordinal, ranked 1 to 9 across age brackets), and clinical position level (ordinal, ranked 1 to 4 among physicians). Country was included as a random intercept, to account for country-level clustering. In addition, institutions were included as a random intercept nested within country, to account for clustering of respondents from the same institution. Clinical position was coded in rank order of professional seniority (1, assistant physician, through 4, medical director). Each coefficient is interpreted as the association per one-category increase in clinical position, or per one-level increase in seniority. The outcome variables were the threshold for initiating prone positioning (ordinal, 1 to 4, ranging from ≤100 to ≤300 mmHg), positioning frequency for full prone positioning (ordinal, 1 to 5, ranking from *never* to *always*) and session duration (continuous, measured in hours). All ordinal predictors were tested for linear trends. Respondents identifying as being in nursing or ‘other’ professional groups were excluded from the clinical position predictor, as were responses of ‘other’ threshold choice, which could not be reliably modeled. Collinearity among predictor variables was assessed using variance inflation factors ( threshold < 5 indicates acceptable collinearity).

A two-sided *p*-value < 0.05 was considered statistically significant. All post hoc analyses were exploratory; *p*-values were not adjusted for multiple testing and should be interpreted descriptively. All statistical analyses and visualization were performed using R (version 4.5.2, R Foundation for Statistical Computing, Vienna, Austria). The map of Europe with the geographic distribution of survey respondents was created by using mapchart.net.

## 3. Results

### 3.1. Respondent Characteristics

After the exclusion of responses with an incomplete characteristics section and responses from outside of Europe, we had 451 fully analyzable responses (Figure S1). Of these, 438 respondents provided valid postal codes that could be reliably mapped to 348 institutions. Respondents were predominantly male senior physicians working in public, often academic hospitals, in small-to-medium medical–surgical ICUs (Table S1). Respondents came from 28 countries, 12 of which contributed fewer than five respondents. Italy (129/451, 28.6%), Germany (83/451, 18.4%) and Austria (52/451, 11.5%) together accounted for 58.5% (Figure S2).

### 3.2. Prone Positioning Practices, Initiation Thresholds and Factors to Determine the Response

Prone positioning was reported to be used by most respondents (446/451, 98.9%), with most indicating 24-hour availability (426/446, 95.5%) (Table S1). The most frequently cited reasons for withholding prone positioning were spinal instability, impending cardiac arrest, and recent (<48 h) major abdominal or thoracic surgery (Table 1).

Initiation thresholds for prone positioning based on PaO2/FiO2 ratios varied substantially among the respondents. More than half (247/446, 55.4%) reported initiating prone positioning at a PaO2/FiO2 ratio ≤ 150 mmHg, whereas fewer than one third of the respondents (120/446, 26.9%) indicated using a threshold ≤ 100 mmHg.

Changes in the PaO_2_/FiO_2_ ratio, driving pressure, and compliance of the respiratory system were most frequently rated as factors used to determine the response to prone positioning (Table 1).

### 3.3. Prone Positioning Strategies and Duration of Prone Positioning

**Figure 1:**
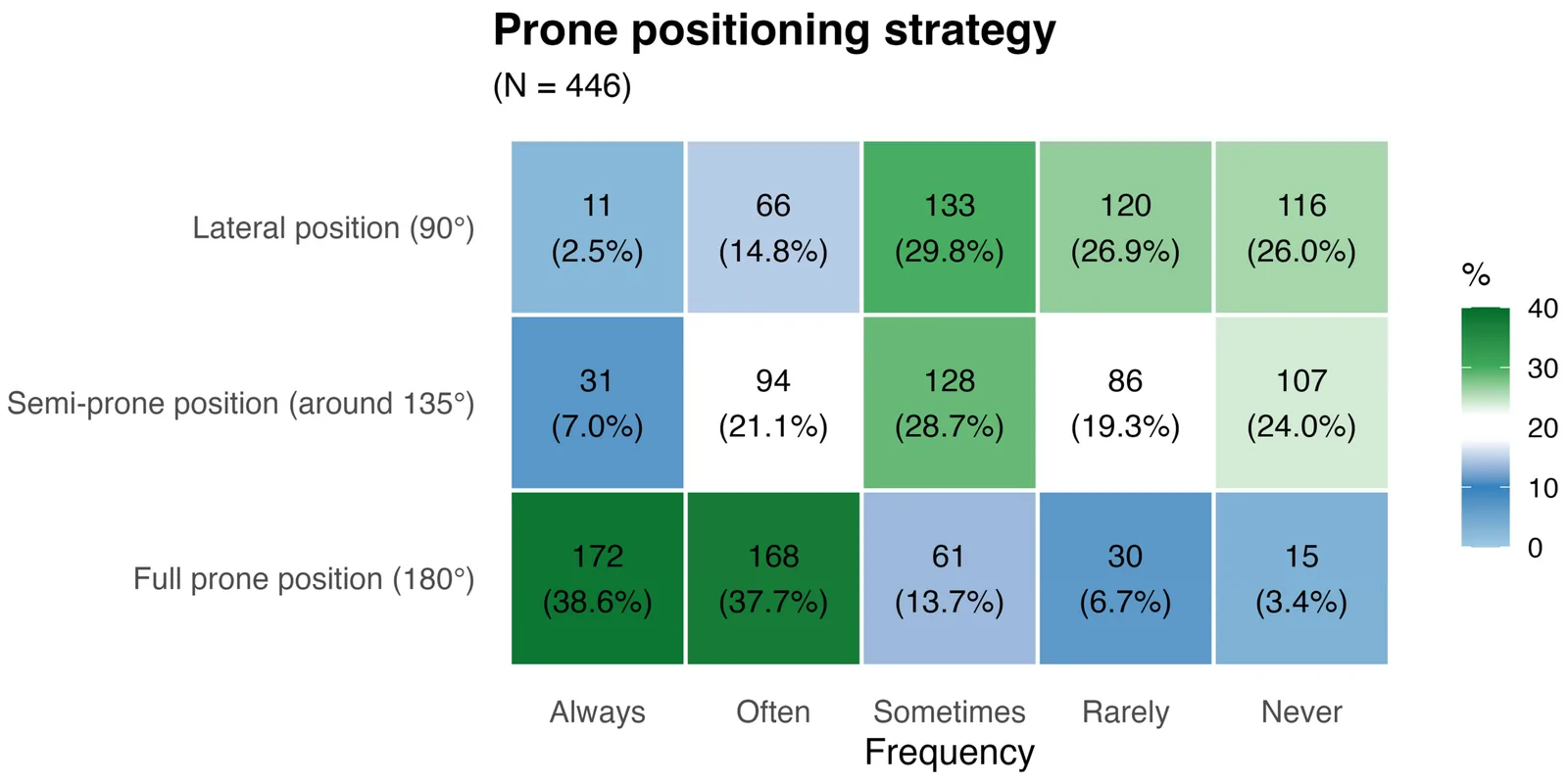
A heatmap showing prone positioning strategies reported by respondents (N = 446). Rows indicate prone positioning strategies, ranging from lateral (90°) to full prone positioning (180°). Columns represent the reported frequency of use, ranging from ‘always’ to ‘never’. Each cell displays the number (%) of respondents within each positioning category. Color indicates the proportion of respondents (blue, lower percentages; green, higher percentages).

The reported prone positioning strategy, whether full prone (180°), semi-prone (135°) or lateral (90°), varied considerably among the respondents (Figure 1). Nearly 80% indicated that they *often* or *always* applied full prone positioning, whereas alternative positioning strategies (semi-prone or lateral) were reported less frequently. About half of respondents (213/446, 47.8%) reported maintaining patients in prone position for 16 h per session. The next most reported durations were 12 h (76/446, 17.0%) and 8 h (35/446, 7.8%).

### 3.4. Use of Awake Prone Positioning Prior to Invasive Ventilation

The use of prone positioning in non-intubated patients varied widely (Table S1). Routine use of awake prone positioning was uncommon (43/446, 9.6%), while 254/446 (57.0%) reported occasional use. One third (149/446, 33.4%) reported not using it in their patients.

### 3.5. Post Hoc Analysis

Fewer than half of the respondents reported practice fully consistent with the ESICM guideline, and only a minority reported practice that was fully consistent with the ATS guideline (Table S5). Concordance for reported initiation thresholds was higher with the ESICM guideline, whereas concordance for the reported duration of prone positioning was slightly higher with the ATS guideline. Reported practice was most often consistent with the ESICM guideline recommendation in Germany; awake prone positioning was also used most often in that country (Table S6). Older age was associated with milder initiation thresholds, larger ICU capacity and higher clinical position were associated with more frequent use of full prone positioning, and both age and clinical position were associated with session duration (shorter with age, longer with seniority). For session duration, residual diagnostics showed approximately symmetric residuals, and the ordinal sensitivity analysis gave results in the same direction (Table S7). Country-level variation was present across all models (Tables S2–S4).

## 4. Discussion

This survey provides several important insights into current prone positioning practices among European clinicians actively managing ARDS: (1) prone positioning was commonly used by the respondents, though reported practice varied markedly; (2) PaO_2_/FiO_2_ thresholds for initiating prone positioning differed substantially between respondents; and (3) full prone positioning was the predominant strategy, applied *always* or *often* by more than three-quarters of the respondents, while semi-prone and lateral positioning were used considerably less often. Reported practice appeared to vary between countries; however, as the sample was not geographically balanced, country-level differences should be interpreted with caution and not as representative of national practice.

In our survey, prone positioning was commonly reported among respondents actively managing ARDS, which is in line with current guideline recommendations [20]. This contrasts with a series of earlier reports that were published after the PROSEVA trial and during the COVID-19 era, although differences in study population and methods preclude direct comparison. In the ‘Large observational study to UNderstand the Global impact of Severe Acute respiratory FailurE’ (LUNG SAFE) [23], prone positioning was applied in only a minority of patients, despite strong evidence supporting its benefit. A subsequent secondary analysis of LUNG SAFE found higher utilization in high-income countries compared with middle- and low-income countries [24], suggesting that local resources and expertise may influence implementation. Observational data from the ‘ARDS Prone Position Network’ (APRONET) study [25], conducted shortly after the landmark PROSEVA trial [5], similarly demonstrated increased adoption, though use remained inconsistent across settings. The COVID-19 pandemic substantially accelerated adoption of prone positioning for both intubated and non-intubated patients, although adoption started declining in the post-COVID-19 era [17].

Prone positioning was reported by almost all respondents, which may in part reflect enrichment of our sample for clinicians with a particular interest in ARDS management. Among these clinicians, the relevant clinical question is therefore less *whether* prone positioning is adopted than *how* it is implemented at the bedside, a gap our data are positioned to address by characterizing the practice patterns reported by clinicians from European ICUs. Beyond adoption itself, our survey provides new information on initiation thresholds, degree of positioning, and duration, dimensions not captured by prior studies. In the post hoc analysis, older age was associated with milder initiation thresholds, and larger ICU size and higher clinical position were associated with more consistent use of full prone positioning. These associations are exploratory. Possible explanations, such as accumulated experience or greater resources and expertise in larger ICUs, were not measured and remain speculative. Age and clinical position were also associated with session duration, though the effect was small and likely not clinically meaningful.

PaO_2_/FiO_2_ thresholds for initiating prone positioning varied widely across the surveyed European respondents. Over half of respondents initiated prone positioning at PaO_2_/FiO_2_ ≤ 150 mmHg, consistent with the ESICM 2023 guideline [20], while nearly one third used ≤100 mmHg, aligning with the more conservative American Thoracic Society 2017 guideline [18]. A small minority (8/446, 1.8%) reported initiating prone positioning at ≤300 mmHg. This does not imply that prone positioning at this threshold is incorrect, rather that its mortality benefit has not been established in mild ARDS in the same way as in moderate to severe ARDS. This question is currently being investigated in the ongoing ‘Effect of PRone positioning on mortality in patients with MILD to moderate ARDS’ (PROMILD) trial (NCT05056090) [26].

Most participants reported using full prone positioning rather than employing alternative positioning strategies (semi-prone or lateral) and a cycling duration mostly between 12 and 16 h. To our knowledge, only one randomized clinical trial has directly compared full with semi-prone positioning [6], showing a benefit for the full prone positioning group at the cost of more frequent facial edema. Current guidelines offer no clear recommendation on the positioning angle [18,19,20].

Approximately 10% of respondents reported routine use of awake prone positioning despite its absence from most guideline recommendations [18,19,20]. Our survey did not differentiate between ARDS and COVID-19–ARDS (C–ARDS) populations, although this distinction is clinically important as the evidence base for awake prone positioning differs substantially between these conditions. The ESICM guidelines support its use in C–ARDS [20], where initial evidence demonstrated a reduction in intubation risk [27]. However, more recent evidence has now demonstrated additional mortality reduction, particularly with a longer daily duration of awake prone positioning [28]. Evidence in non-COVID-19 ARDS remains limited, which may explain the heterogeneous implementation patterns observed in our cohort and the ongoing trials evaluating its efficacy in broader ARDS populations [14,29].

Because respondents were self-selected through networks with a specific focus on ARDS and mechanical ventilation, our findings may overestimate the use of prone positioning and should not be extrapolated to unselected European ICU practices. This selection most likely biases all main findings in the same direction, towards more frequent use of prone positioning, such that practice in unselected European ICUs may differ from the practice reported in our cohort.

In the survey, some response options were not operationally defined; for example, ‘septic shock’ did not distinguish between stabilized and refractory shock, ‘very high catecholamine requirement’ lacked a predefined dose or threshold, and the specified thresholds for acidosis and hypercapnia represented severe physiological derangement rather than established contraindications. Moreover, since job titles may differ between European healthcare systems, the clinical position hierarchy may not be fully equivalent across countries, although entering it as a categorical variable did not change the results.

This survey has several other limitations. The main limitation is inherent to the design, as respondents reported their general practice and stated protocols rather than providing data on actual patient-level implementation. Therefore, actual bedside practice may differ from reported intention. Also, since we did not collect national or institutional guidelines on prone positioning, we could only compare reported practice with two international guidelines [18,20]. In addition, although responses were obtained from many respondents, the sample is not fully representative of all European ICUs and should be interpreted as reflecting routine clinical practice among clinicians actively managing ARDS, rather than a definitive overview of European ICUs. The near-universal reported use of prone positioning and high self-reported 24/7 availability should therefore be interpreted as characteristic of this respondent population rather than of contemporary European ICU practice as a whole. Because the survey was distributed as an open link, the number of clinicians reached is unknown and a response rate could not be calculated; the extent of non-response cannot be assessed. Because the sampling frame was undefined, and the sampling unit was the individual clinician, our results do not permit estimation of the prevalence of prone positioning practices among European ICUs. Furthermore, we would like to emphasize that there was a marked response density peak where our coordinating and sub-investigators are located (Hamburg, Trento, and Genova) and that the two countries with the highest response share (Italy and Germany) are also the countries hosting our lead and sub-investigators. As a result, the sample is not geographically balanced: Italy, Germany and Austria are substantially overrepresented, whereas many countries contributed only a few respondents, and country-level findings should not be interpreted as representative of national practice. This further limits the generalizability, because responses within some countries may themselves be concentrated around specific institutions or regions rather than reflecting national practice. Finally, the post hoc analyses were exploratory and involved multiple comparisons without adjustment, so individual associations should only be considered hypothesis-generating.

In conclusion, among self-selected clinicians working in European ICUs, prone positioning was commonly reported, though reported practice varied with respect to initiation thresholds, positioning strategy and duration and was only partly concordant with current guideline recommendations. These findings identify specific areas where practice diverges and where current guidance and evidence remain incomplete.

## Figures and Tables

**Table 1:** Initiation thresholds, reasons to withhold, and importance of factors for determining response (N = 446).

*Thresholds for initiation of prone positioning,* n (%)				
PaO_2_/FiO_2_ ≤ 100 mmHg				120 (26.9)
PaO_2_/FiO_2_ ≤ 150 mmHg				247 (55.4)
PaO_2_/FiO_2_ ≤ 200 mmHg				51 (11.4)
PaO_2_/FiO_2_ ≤ 300 mmHg				8 (1.8)
other				20 (4.5)
*Reasons to withhold prone positioning,* n (%) *				
Absolute contraindications				
spinal instability				338 (75.8)
impending cardiac arrest				336 (75.3)
Relative contraindications/individualized assessments				
very high catecholamine requirement				150 (33.6)
septic shock				47 (10.5)
Risk factors				
recent major abdominal or thoracic surgery (<48 h)				258 (57.8)
severe obesity (BMI ≥ 40 kg/m^2^)				79 (17.7)
severe coagulopathy or thrombocytopenia without active bleeding				25 (5.6)
Conditions requiring stabilization/optimization				
severe hypercapnia (PaCO_2_ > 60 mmHg)				23 (5.2)
severe respiratory acidosis (pH < 7.25)				19 (4.3)
severe metabolic acidosis (pH < 7.25)				13 (2.9)
other				30 (6.7)
*Importance of factors for determining response to prone positioning*				
change in, n (%)	High	Moderate	Low	None
PaO_2_/FiO_2_	342 (76.7)	82 (18.4)	18 (4.0)	4 (0.9)
PaO_2_/(FiO_2_ × MAP)	29 (6.5)	88 (19.7)	115 (25.8)	214 (48.0)
SpO_2_/FiO_2_	68 (15.2)	119 (26.7)	129 (28.9)	130 (29.1)
AaDO_2_	31 (7.0)	95 (21.3)	154 (34.5)	166 (37.2)
PaCO_2_	116 (26.0)	197 (44.2)	102 (22.9)	31 (7.0)
V_T_	96 (21.5)	222 (49.8)	101 (22.6)	27 (6.1)
ΔP	235 (52.7)	170 (38.1)	33 (7.4)	8 (1.8)
C_RS_	203 (45.5)	184 (41.3)	51 (11.4)	8 (1.8)
Pplat	165 (37.0)	191 (42.8)	71 (15.9)	19 (4.3)
FiO_2_	196 (43.9)	178 (39.9)	63 (14.1)	9 (2.0)
PAP	16 (3.6)	72 (16.1)	133 (29.8)	225 (50.4)
chest image	107 (24.0)	160 (35.9)	133 (29.8)	46 (10.3)
clinical impression	171 (38.3)	167 (37.4)	85 (19.1)	23 (5.2)

Data are n (%) of 446 respondents who reported using prone positioning. * More than one answer was possible. Abbreviations: BMI, body mass index; PaCO_2_, partial pressure of carbon dioxide; PaO_2_, partial pressure of oxygen; FiO_2_, fraction of inspired oxygen; MAP, mean airway pressure; SpO_2_, oxygen saturation; AaDO_2_, alveolar–arterial oxygen difference; C_RS_, compliance of the respiratory system; V_T_, tidal volume; ΔP, driving pressure; Pplat, plateau pressure; PAP, pulmonary artery pressure.

## Data Availability

De-identified data, with institutions replaced by coded identifiers, can be made available by the corresponding author upon reasonable request and subject to approval by the University Medical Centre Hamburg-Eppendorf.

## Supplementary Materials

The following supporting information can be downloaded at https://www.mdpi.com/article/10.3390/jcm15208082/s1, Methods S1: STROBE statement–checklist for items for cross–sectional studies; Methods S2: Checklist for Reporting Results of Internet E–Surveys (CHERRIES); Methods S3: Survey questionnaire items analyzed in this report; Figure S1: Flow diagram of survey respondents included in and excluded from the analysis. Of 592 eligible responses, 451 complete responses from European clinicians were included in the final analysis. Reasons for exclusion are depicted on the right; Figure S2: Geographic distribution of CUPIDO survey respondents across Europe. Countries are shaded according to the number of respondents. The largest contributions were from Italy (28.6%), Germany (18.4%), and Austria (11.5%). Countries without respondents are shown in grey; Table S1: Participants, Hospital and ICU Characteristics, and use of Prone Positioning (N = 451); Table S2: Model 1: PaO_2_/FiO_2_ threshold for initiating prone positioning, ordinal mixed–effect model, country and institution nested within country as random intercept; Table S3: Model 2: Full prone positioning frequency, ordinal mixed–effect model, country and institution nested within country as random intercept; Table S4: Model 3: Minimum duration of one prone positioning cycle, linear mixed–effect model, country and institution nested within country as random intercept; Table S5: Concordance of Reported Practice with International Guideline Recommendations; Table S6: Concordance with Recommendations in the ESICM Guideline and Use of Awake Prone Positioning; Table S7: Session duration: linear model and ordinal sensitivity model (N = 425).

## Endorsement

This study was endorsed by the European Society of Intensive Care Medicine.

## Abbreviations

ARDS	acute respiratory distress syndrome
ATS	American Thoracic Society
C–ARDS	COVID-19 ARDS
COVID-19	coronavirusdisease 2019
ESICM	European Society of Intensive Care Medicine
FiO_2_	fraction of inspired oxygen
ICU	intensive care unit
PaO_2_	partial pressure of oxygen in arterial blood
