# Supplementary material for: Current Practices of Prone Positioning in ARDS Reported by European ICU Clinicians (CUPIDO)—A Cross-Sectional Questionnaire-Based Survey
Source: J Clin Med. 2026 Oct 18;15(20):8082. doi: 10.3390/jcm15208082 (PMC13705875; doi:10.3390/jcm15208082)
Supplement: Supplementary file 1 [file jcm-15-08082-s001.zip › jcm-4551286-supplementary.pdf]

SUPPLEMENT TO:

Current Practices of Prone Positioning in ARDS Reported by European ICU Clinicians (CUPIDO)–A Cross–Sectional Questionnaire–Based Survey

Fabian Gleibs[1]*, Markus Haar[1]*, Denise Battaglini[2,3], Silvia de Rosa[4,5], Sergio Lassola[4], Aneta Volfová[1], Lise Piquilloud[6], Stefan Kluge[1], Marcus J. Schultz[7–10], Kevin Roedl[1]

Table of Contents

Methods S1. 3

Methods S2. 7

Methods S3. 11

Figure S1. 16

Figure S2. 17

Table S1. 18

Table S2. 20

Table S3. 21

Table S4. 22

Table S5. 23

Table S6. 24

Table S7. 25

Methods S1. STROBE statement–checklist for items for cross–sectional studies

Section / Topic	Item	Recommendation	Where reported in the manuscript	Page
**Title and abstract**				
Title and abstract	1	(a) Indicate the study's design with a commonly used term in the title or the abstract	Title ("A Cross–Sectional Questionnaire–Based Survey"); Abstract, Methods	1, 3
		(b) Provide in the abstract an informative and balanced summary of what was done and what was found	Abstract (structured: Background, Methods, Results, Conclusion)	3–4
**Introduction**				
Background/ rationale	2	Explain the scientific background and rationale for the investigation being reported	Introduction, paragraphs 1–3 (PROSEVA, external validity, guideline differences)	5–6
Objectives	3	State specific objectives, including any prespecified hypotheses	Introduction, last paragraph (aim and hypothesis)	5–6
**Methods**				
Study design	4	Present key elements of study design early in the paper	Methods, Design (cross–sectional online survey; registration NCT06714201)	7
Setting	5	Describe the setting, locations, and relevant dates, including periods of recruitment, exposure, follow–up, and data collection	Methods, Survey dissemination (open link via ESICM, PROVE Network, ESAIC, national societies and professional networks; 7 March – 31 August 2025)	8
Participants	6	(a) Give the eligibility criteria, and the sources and methods of selection of participants	Methods, survey development and content; Participants and eligibility	8–9
Variables	7	Clearly define all outcomes, exposures, predictors, potential confounders, and effect modifiers. Give diagnostic criteria, if applicable	Methods, Outcomes (initiation threshold, positioning strategy, session duration); Statistical analysis (ICU size, age, clinical position; coding)	9–10

Data sources/ measurement	8	For each variable of interest, give sources of data and details of methods of assessment (measurement). Describe comparability of assessment methods if there is more than one group	Methods, Survey development and content (panel, face/content validity, pilot); questionnaire items analyzed in this report in Supplement (Methods S3)	7–8
Bias	9	Describe any efforts to address potential sources of bias	Methods, Statistical analysis (restriction to completed characteristics section to limit duplicates; institutions as nested random intercept); Discussion, Limitations (self–selection, non–response, over–represented countries)	9–10, 17–19
Study size	10	Explain how the study size was arrived at	No formal sample size calculation was performed; all complete responses from European respondents received during the survey period were included	–
Quantitative variables	11	Explain how quantitative variables were handled in the analyses. If applicable, describe which groupings were chosen and why	Methods, Statistical analysis (ordinal scores for ICU size 1–5, age 1–9, clinical position 1–4; outcome coding; linearity tested); Tables S2–S4 footnotes	10
Statistical methods	12	(a) Describe all statistical methods, including those used to control for confounding	Methods, Statistical analysis (cumulative link and linear mixed models; random intercepts for country and institution nested within country; VIF)	9–11
		(b) Describe any methods used to examine subgroups and interactions	Methods, Statistical analysis (post–hoc comparison with ESICM 2023 and ATS 2017 guidelines; Tables S5–S6)	9
		(c) Explain how missing data were addressed	Methods, Statistical analysis (no imputation; denominators per item in table footnotes)	9

		(d) If applicable, describe analytical methods taking account of sampling strategy	Not applicable (open, non–probability survey; no weighting)	–
		(e) Describe any sensitivity analyses	Tables S2–S4 footnotes (proportional odds, merged categories, categorical coding); Table S7 (ordinal model for session duration)	10, 15, Suppl.
**Results**				
Participants	13	(a) Report numbers of individuals at each stage of study–e.g., numbers potentially eligible, examined for eligibility, confirmed eligible, included in the study, completing follow–up, and analysed	Results, paragraph 1; Figure S1 (592 responses → 451 included; 446 using prone positioning)	12, Suppl.
		(b) Give reasons for non–participation at each stage	Figure S1 (no country given, outside Europe, stopped before first content question)	Suppl.
		(c) Consider use of a flow diagram	Figure S1	Suppl.
Descriptive data	14	(a) Give characteristics of study participants (e.g., demographic, clinical, social) and information on exposures and potential confounders	Results, paragraph 1; Table S1; Figure S2 (countries)	12, Suppl.
		(b) Indicate number of participants with missing data for each variable of interest	Denominators reported in each table and table footnotes	9; Suppl.
Outcome data	15	Report numbers of outcome events or summary measures	Results; Table 1; Figure 1	12–15, Table 1, Figure 1
Main results	16	(a) Give unadjusted estimates and, if applicable, confounder–adjusted estimates and their precision (e.g., 95% confidence interval). Make clear which confounders were adjusted for and why they were included	Results, Post–hoc analysis; Tables S2–S4 (odds ratios / β with 95% CI)	15, Suppl.

		(b) Report category boundaries when continuous variables were categorized	Methods, Statistical analysis; Tables S2–S4 and S7	10, Suppl.
		(c) If relevant, consider translating estimates of relative risk into absolute risk for a meaningful time period	Not applicable	–
Other analyses	17	Report other analyses done–e.g., analyses of subgroups and interactions, and sensitivity analyses	Tables S5–S7; footnotes of Tables S2–S4	15, Suppl.
**Discussion**				
Key results	18	Summarise key results with reference to study objectives	Discussion, paragraph 1	16
Limitations	19	Discuss limitations of the study, taking into account sources of potential bias or imprecision. Discuss both direction and magnitude of any potential bias	Discussion, Limitations (self–reported practice, self–selection and its direction, unknown response rate, sampling unit, geographic imbalance, response options not operationally defined, exploratory post hoc analyses)	18–20
Interpretation	20	Give a cautious overall interpretation of results considering objectives, limitations, multiplicity of analyses, results from similar studies, and other relevant evidence	Discussion, paragraphs 2–6; Conclusion	16–20
Generalisability	21	Discuss the generalisability (external validity) of the study results	Discussion, Limitations (not representative of European ICU practice; country–level findings not representative of national practice)	18–20
**Other information**				
Funding	22	Give the source of funding and the role of the funders for the present study and, if applicable, for the original study on which the present article is based	Funding statement (no external funding)	4, 27

Methods S2. Checklist for Reporting Results of Internet E–Surveys (CHERRIES)

Item category	Checklist item	CUPIDO	Where reported	Page
**Design**				
Design	Describe survey design	Cross–sectional, open, web–based survey of healthcare professionals (primarily physicians) working in European ICUs; convenience (non–probability) sample without defined sampling frame	Methods, Design; Participants and eligibility	7–9
**IRB approval and informed consent process**				
IRB approval	Mention whether the study has been approved by an IRB	Formal ethical review not sought (no intervention, no patient data, no data attributable to identifiable individuals; German professional code for physicians)	Methods, Design; Ethics approval statement	7, 27
Informed consent	Describe the informed consent process (length of survey, data storage, investigator, purpose)	Consent page at start of the questionnaire (purpose, investigators, voluntary participation, approx. 20 minutes, data handling); proceeding constituted consent	Methods, Design; Supplement (consent page)	7, 27, Suppl.
Data protection	If any personal information was collected or stored, describe mechanisms to protect it	No names, contact details or patient data collected; postal codes used only to identify institutions, results reported in aggregate	Methods, Design	7
**Development and pre–testing**				
Development and testing	State how the survey was developed, including whether usability and technical functionality were tested	Developed de novo by seven senior ICU physicians from five centers; face and content validity by panel consensus; pilot test by two physicians not involved in development, minor wording changes	Methods, Survey development and content	7–8
**Recruitment process and description of the sample having access to the questionnaire**				

Open survey versus closed survey	Open survey (any visitor) or closed survey (invited only, password)	Open survey (open link)	Methods, Survey dissemination	8
Contact mode	Was initial contact made on the Internet?	Yes – electronic distribution via societies, networks and professional contacts	Methods, Survey dissemination	8
Advertising the survey	How/where was the survey announced or advertised?	ESICM, PROVE Network, ESAIC, national intensive care societies and professional networks; how respondents became aware of the survey is reported	Methods, Survey dissemination, Table S1	8, Suppl.
**Survey administration**				
Web/E–mail	Type of e–survey and how responses were captured	Web–based questionnaire on SurveyMonkey; responses captured automatically	Methods, Survey development and content	8
Context	Describe the website on which the survey was posted	Not applicable (no hosting website; distributed as a direct link)	–	–
Mandatory/voluntary	Was it mandatory or voluntary?	Voluntary; no question was mandatory	Methods, Design, Survey development and content	7–8
Incentives	Were any incentives offered?	No	Methods, Design	7
Time/Date	In what timeframe were the data collected?	7 March – 31 August 2025	Methods, Survey dissemination	8
Randomization of items or questionnaires	Were items randomized or alternated?	No randomization	–	–
Adaptive questioning	Were certain items displayed conditionally on earlier responses?	Yes – respondents not using prone positioning were automatically directed to the next questionnaire domain	Methods, Survey development	8
Number of items	Number of questionnaire items per page	1	Supplement (Methods S3, questionnaire)	Suppl.

Number of screens (pages)	Over how many pages was the questionnaire distributed?	24	Supplement (Methods S3, questionnaire)	Suppl.
Completeness check	Were completeness checks used?	No forced responses; no question was mandatory	Methods, Participants and eligibility	8
Review step	Were respondents able to review and change their answers (e.g., Back button)?	Yes	–	–
**Response rates**				
Unique site visitor	How were unique visitors determined?	Not determined	–	–
View rate	Unique survey visitors / unique site visitors	Not available (open link; number of clinicians reached unknown)	Discussion, Limitations	19
Participation rate	Unique visitors who agreed to participate / unique first survey page visitors	Not available	Discussion, Limitations	19
Completion rate	Users who finished the survey / users who agreed to participate	Numbers at each stage shown in the flow diagram	Results, paragraph 1; Figure S1	12, Suppl.
**Preventing multiple entries from the same individual**				
Cookies used	Were cookies used to identify multiple entries?	No	–	–
IP check	Was the IP address used to identify multiple entries?	No	–	–
Log file analysis	Were other techniques used to identify multiple entries?	Restriction to responses with a completed characteristics section to minimize duplicate entries	Methods, Statistical analysis	9

Registration	In closed surveys, how did users log in?	Not applicable (open survey)	–	–
**Analysis**				
Handling of incomplete questionnaires	Were only completed questionnaires analysed? Were partial questionnaires included?	Included if characteristics section completed and either no prone positioning reported or prone positioning section completed through its final question; missing items not imputed, denominators per item	Methods, Survey developent; Statistical analysis	8–9
Questionnaires submitted with an atypical timestamp	Were completion times used to exclude responses?	No	–	–
Statistical correction	Were weighting or propensity scores used to adjust for the non–representative sample?	No weighting or statistical correction	Discussion, Limitations	17–19

Methods S3.

Survey questionnaire items analyzed in this report

Thank you for participating in our survey that will help to inform about the current practices of ARDS treatment. It will take you about 20 minutes to complete the survey. No personal data will be collected.

This study was conceived as collaborative project of the University of Hamburg (Department of Intensive Care Medicine, UKE Hamburg–Eppendorf), the University of Trento (Center for Medical Sciences, Santa Chiara Hospital), the University of Genova, and the University of Amsterdam (Department of Intensive Care Medicine, Amsterdam UMC) and is part of the PROtective VEntilation (PROVE) Network.

This survey complies with the General Data Protection Regulation (GDPR) of the European Union to ensure the confidentiality and protection of your data. Participation in this survey is voluntary, and no personal identifiable information will be collected. All responses will be anonymised and used solely for the purposes of this research project to evaluate current practices in the management of ARDS in European ICUs. By proceeding, you consent to the processing of your responses for research purposes

Personal Information

What is your age?
- ☐ ≤ 29 years
- ☐ 30 to 34 years
- ☐ 35 to 39 years
- ☐ 40 to 44 years
- ☐ 45 to 49 years
- ☐ 50 to 54 years
- ☐ 55 to 59 years
- ☐ 60 to 64 years
- ☐ ≥ 65 years
- ☐ Prefer not to say

What is your gender?
- ☐ Female
- ☐ Male
- ☐ Non–binary
- ☐ Prefer not to say

What is your current clinical position level?
- ☐ Medical Director
- ☐ Senior Physician
- ☐ Attending (Consultant, Medical Specialist)
- ☐ Assistant Physician (Registrar, Junior Doctor, Intern)
- ☐ Nursing
- ☐ Other (please specify)

How were you made aware of this survey? (You can select more than one answer)
- ☐ Institutional or organizational newsletter
- ☐ Professional network or colleague recommendation
- ☐ Medical conference or event announcement
- ☐ Social media (e.g., LinkedIn, Twitter, Facebook)
- ☐ Website or online forum
- ☐ Other (please specify)

Hospital and Department Characteristics

In which country do you work?

____

What is the zip code of your hospital/institution?

____

What kind of hospital are you working for?
- ☐ Public
- ☐ Private

- ☐ Military
- ☐ Unsure
- ☐ Other (please specify)

What is the level of care that is provided by your hospital?
- ☐ Academic/University hospital
- ☐ Non–academic/non–university hospital
- ☐ Prefer not to say

Which conditions are commonly treated in your ICU?
(You can select more than one answer)
- ☐ Medical
- ☐ Surgical
- ☐ Neurological/Neurosurgical
- ☐ Pediatric/Neonatal
- ☐ Other (please specify)

What is the approximate number of ICU beds in your hospital?
(not including intermediate care)
- ☐ < 10
- ☐ 10–20
- ☐ 21–30
- ☐ 31–40
- ☐ > 40

Prone Positioning
Regarding the following questions, prone position cycles are defined as the individual periods during which patients are proned. Prone position therapy describes the period from the beginning of the first prone position cycle to the end of the last prone position cycle.

Does your ICU perform prone position therapy to treat ARDS?
- ☐ Yes
- ☐ No

Why are you not performing prone position in your ICU?
(You can select more than one answer)
- ☐ Concerns about patient safety (e.g., risk of complications or injuries)
- ☐ Unfamiliarity with current guidelines or lack of confidence in their efficacy
- ☐ No perceived benefit or belief that other treatments are more effective
- ☐ High patient acuity or complexity of cases makes prone positioning unfeasible
- ☐ Policy or administrative restrictions (e.g., institutional or national guidelines)
- ☐ Logistical challenges in mobilizing patients for prone positioning
- ☐ Lack of training opportunities for staff on prone positioning techniques

As soon as indicated, will prone position therapy be initiated around the clock (including during night)?

☐ Yes
☐ No

When is prone position therapy usually initiated to treat ARDS in your ICU?
☐ PaO2/FiO2 ≤ 300 mmHg
☐ PaO2/FiO2 ≤ 200 mmHg
☐ PaO2/FiO2 ≤ 150 mmHg
☐ PaO2/FiO2 ≤ 100 mmHg
☐ Other (please specify)

Is prone position therapy initiated prior to invasive ventilation (awake proning) in your ICU?
☐ Yes, routinely
☐ Yes, occasionally
☐ No

To what extent are patients usually proned in your ICU?

	Never	Rarely	Sometimes	Often	Always
Complete prone position (180°)	☐	☐	☐	☐	☐
Incomplete/partial prone position (around 135°)	☐	☐	☐	☐	☐
Lateral position (90°)	☐	☐	☐	☐	☐

How many personnel (physicians and nurses) are usually engaged in prone or supine repositioning? (Move the slider)
________________ Number of personnel

Do you continue prone position therapy to reach a minimum number of cycles, even if oxygenation (supine/between cycles) has improved significantly? If there is no threshold move the slider to the leftmost position. This corresponds to a value of '1' in the input field to the right.
________________ (Slider 1–10,Threshold of minimum cycles)

For each prone position cycle, what is the usual minimum duration (in hours) that patients are kept in prone position? (Move the slider)
________________ (Slider 1–24, Duration in hours)

What level of importance do you assign to the following parameters for estimating therapy response (e.g., prone position) in ARDS in your <u>actual</u> clinical practice?

	None	Low	Moderate	High
PaO_2/FiO_2	☐	☐	☐	☐
$PaO_2/(FiO_2$ x MAP)	☐	☐	☐	☐
SpO_2/FiO_2	☐	☐	☐	☐
FiO_2	☐	☐	☐	☐

$AaDO_2$	☐	☐	☐	☐
Decrease in $PaCO_2$	☐	☐	☐	☐
Chest imaging	☐	☐	☐	☐
Compliance of the respiratory system	☐	☐	☐	☐
Tidal volume	☐	☐	☐	☐
Driving pressure	☐	☐	☐	☐
Plateau pressure	☐	☐	☐	☐
Pulmonary artery pressure	☐	☐	☐	☐
Clinical impression	☐	☐	☐	☐

A patient diagnosed with ARDS has a PaO_2/FiO_2 ratio of 100 mmHg before the initiation of prone position therapy. To what value must the PaO_2/FiO_2 ratio increase during prone positioning for you to consider it a relevant response to treatment? (Move the slider)

_________________ (PaO_2/FiO_2)

If prone positioning leads to increased compliance and, consequently, an increase in tidal volume during pressure–controlled ventilation, how does your ICU manage this change in the majority of cases?

- ☐ Accept the increased tidal volume if plateau pressure remains safe
- ☐ Adjust ventilator settings to decrease tidal volume, even if compliance improves
- ☐ Decrease respiratory rate to maintain minute volume
- ☐ Monitor and adjust based on patient–specific tolerance and response
- ☐ No specific strategy
- ☐ Other (please specify)

What are the predominant clinical circumstances in your ICU under which prone positioning is withheld? (You can select more than one answer)

- ☐ spinal instability
- ☐ impending cardiac arrest
- ☐ recent major abdominal or thoracic surgery (< 48 h)
- ☐ very high catecholamine requirement
- ☐ severe obesity (BMI ≥ 40 kg/m^2)
- ☐ septic shock
- ☐ severe coagulopathy or thrombocytopenia without active bleeding
- ☐ severe hypercapnia ($PaCO_2$ > 60 mmHg)
- ☐ severe respiratory acidosis (pH < 7.25)
- ☐ severe metabolic acidosis (pH < 7.25)
- ☐ other

Is ECMO therapy available in your ICU?

- ☐ Yes

☐ No

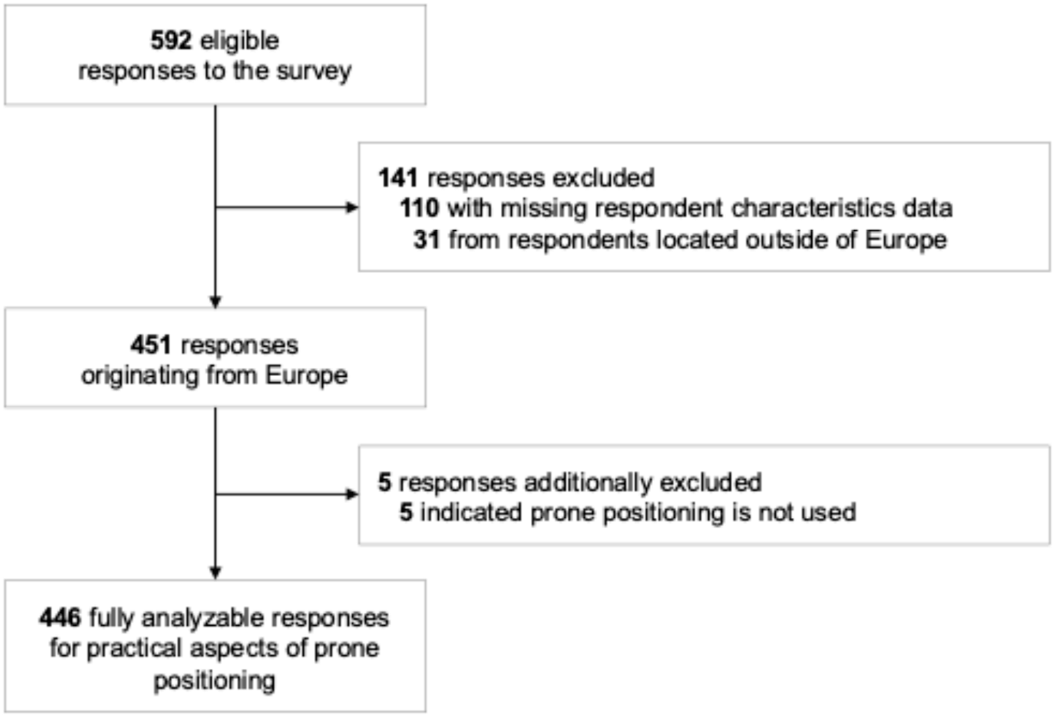

Figure S1. Flow diagram of survey respondents included in and excluded from the analysis. Of 592 eligible responses, 451 complete responses from European clinicians were included in the final analysis. Reasons for exclusion are depicted on the right.

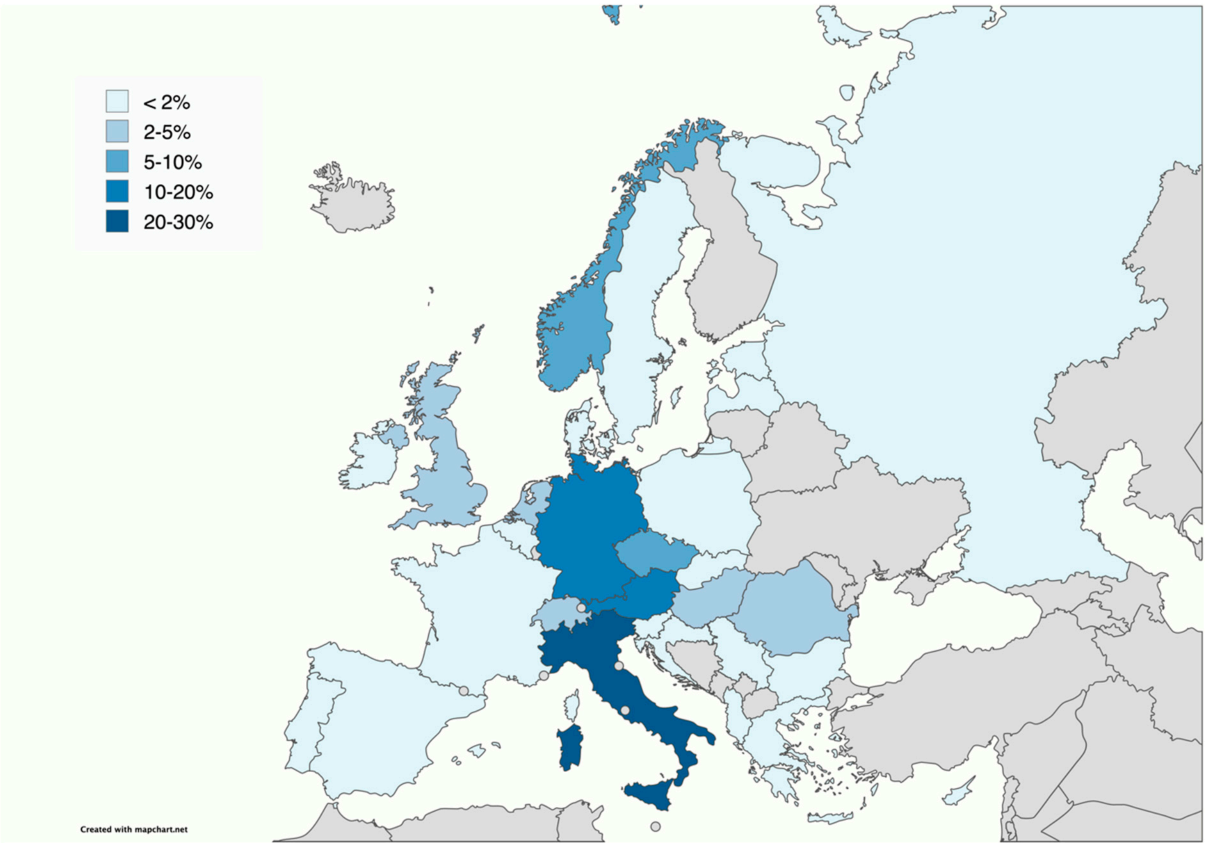

Figure S2. Geographic distribution of CUPIDO survey respondents across Europe. Countries are shaded according to the number of respondents. The largest contributions were from Italy (28.6%), Germany (18.4%), and Austria (11.5%). Countries without respondents are shown in grey.

Table S1. Participants, Hospital and ICU Characteristics, and use of Prone Positioning (N = 451)

Participants characteristics	
sex, male, n (%)	298 (66.1)
age categories in years, n (%)	
≤ 29 years	18 (4.0)
30–34	61 (13.5)
35–39	75 (16.6)
40–44	87 (19.3)
45–49	75 (16.6)
50–54	40 (8.9)
55–59	40 (8.9)
60–64	39 (8.6)
≥ 65	15 (3.3)
prefer not to say	1 (0.2)
professional status, n (%)	
medical director	61 (13.5)
attending (consultant, medical specialist)	174 (38.6)
senior physician	158 (35.0)
assistant physician (registrar, junior doctor, intern)	37 (8.2)
nursing	12 (2.7)
other	9 (2.0)
awareness of survey, n (%)*	
professional network or colleague recommendation	154 (34.1)
institutional or organizational newsletter	223 (49.4)
medical conference or event announcement	3 (0.7)
social media (e.g., LinkedIn, Twitter, Facebook)	29 (6.4)
website or online forum	30 (6.7)
other	24 (5.3)
Hospital and ICU characteristics	
type of hospital, n (%)	
public	407 (90.2)
private	30 (6.7)
military	8 (1.8)
other	6 (1.3)
level of care, n (%)	
academic/university hospital	270 (59.9)
non–academic/non–university hospital	181 (40.1)
patient types treated, n (%)*	
medical	390 (86.5)
surgical	363 (80.5)
neurological/neurosurgical	195 (43.2)
paediatric/neonatal	39 (8.6)
other	21 (4.7)
ICU size categories in beds, n (%)	
< 10	108 (23.9)
10–20	140 (31.0)
21–30	64 (14.2)
31–40	29 (6.4)
> 40	110 (24.4)
Prone Positioning Practice	

prone positioning is practiced, yes, n (%)	446 (98.9)
around the clock, yes, n (%) †	426 (95.5)
in non–intubated patients, yes, n (%) †	297 (66.6)

Abbreviations: ICU, intensive care unit; †, Denominator = 446 (prone positioning users) instead of 451 (full sample), *more than one answer was possible

Table S2. Model 1: PaO_2/FiO_2 threshold for initiating prone positioning, ordinal mixed–effect model, country and institution nested within country as random intercept

Predictor	OR (95% CI)	p–value	VIF
ICU size	1.015 (0.875–1.176)	0.846	1.006
age	1.197 (1.057–1.356)	0.005*	1.352
clinical position	1.056 (0.787–1.416)	0.718	1.359

Country random–intercept variance = 0.428 (SD = 0.654); institution random–intercept variance = 0.372 (SD = 0.610)

N = 406 respondents from 27 countries and 331 clusters (institutions, with respondents without usable postal code treated as separate clusters); physicians using prone positioning who reported their age, excluding other healthcare professionals (e.g. nurses) and respondents who specified 'other' threshold. Ordinal variables: ICU size (1, < 10 beds through 5, > 40 beds), age (1, ≤ 29 years through 9, ≥ 65 years), and clinical position (1, assistant physician through 4, medical director); Model 1 outcome: PaO_2/FiO_2 threshold for initiating prone positioning (1 = ≤ 100 mmHg, 2 = ≤ 150 mmHg, 3 = ≤ 200 mmHg, 4 = ≤ 300 mmHg; higher numbers indicate more conservative/milder hypoxemia thresholds); OR >1 indicates increased odds of selecting a higher threshold; OR <1 indicates increased odds of selecting a lower threshold. Random–intercept variances indicate heterogeneity between countries and between institutions. VIF < 5 indicates acceptable collinearity; Exploratory analysis, p–values not adjusted for multiple testing. *p < 0.05.

The proportional–odds assumption was assessed with likelihood–ratio tests in the corresponding fixed–effects model (global p=0.051; p=0.059 after merging the sparse categories ≤ 200 and ≤ 300 mmHg); odds ratios were virtually unchanged after the merging.

Entering age, ICU size or clinical position as categorical variables instead of linear scores did not improve model fit (likelihood–ratio tests, all p≥ 0.375)

Abbreviations: PaO_2/FiO_2, ratio of arterial oxygen partial pressure to fraction of inspired oxygen; ICU, intensive care unit; OR, odds ratio; CI, confidence interval; VIF, variance inflation factor; SD, standard deviation.

Table S3. Model 2: Full prone positioning frequency, ordinal mixed–effect model, country and institution nested within country as random intercept

Predictor	OR (95% CI)	p–value	VIF
ICU size	1.203 (1.031–1.404)	0.019*	1.005
age	0.949 (0.841–1.072)	0.401	1.355
clinical position	1.385 (1.020–1.879)	0.037*	1.359
Country random–intercept variance = 0.672 (SD = 0.820); institution random–intercept variance = 0.809 (SD = 0.900)			

N = 425 respondents from 27 countries and 340 clusters (institutions, with respondents without a usable postal code treated as separate clusters); physicians using prone positioning who reported their age, other healthcare professionals (e.g. nurses) excluded. Ordinal variables: ICU size (1, < 10 beds through 5, > 40 beds), age (1, ≤ 29 years through 9, ≥ 65 years), and clinical position (1, assistant physician through 4, medical director); Model 2 outcome: Full prone positioning frequency (1 = never, 2 = rarely, 3 = sometimes, 4 = often, 5 = always); OR >1 indicates increased odds of more frequent use; OR <1 indicates decreased odds of frequent use. Random–intercept variances indicate heterogeneity between countries and between institutions. VIF < 5 indicates acceptable collinearity; Exploratory analysis, p–values not adjusted for multiple testing. *p < 0.05.

The proportional–odds assumption was assessed with likelihood–ratio tests in the corresponding fixed–effects model (global p=0.028; p=0.067 after merging the sparse categories ‘never’ and ‘rarely’); odds ratios were virtually unchanged after the merging.

Entering age, ICU size or clinical position as categorical variables instead of linear scores did not improve model fit (likelihood–ratio tests, all p≥ 0.125)

Abbreviations: ICU, intensive care unit; OR, odds ratio; CI, confidence interval; VIF, variance inflation factor; SD, standard deviation.

Table S4. Model 3: Minimum duration of one prone positioning cycle, linear mixed–effect model, country and institution nested within country as random intercept

Predictor	β (95% CI)	p–value	VIF
ICU size	0.198 (−0.079 to 0.475)	0.162	1.005
age	−0.291 (−0.511 to −0.071)	0.010*	1.355
clinical position	0.560 (0.018 to 1.103)	0.044*	1.359

Country random–intercept variance = 2.751 (SD = 1.659); institution random–intercept variance = 2.626 (SD = 1.620); residual variance = 13.427

N = 425 respondents from 27 countries and 340 clusters (institutions, with respondents without a usable postal code treated as separate clusters); physicians using prone positioning who reported their age; other healthcare professionals (e.g. nurses) excluded. Ordinal variables: ICU size (1, < 10 beds through 5, > 40 beds), age (1, ≤ 29 years through 9, ≥ 65 years), and clinical position (1, assistant physician through 4, medical director); Model 3 outcome: Minimum duration of one prone positioning cycle in hours (continuous variable); β represents the change in duration (hours) per one–rank increase in the predictor variable; Positive β indicates longer duration; negative β indicates shorter duration. Random–intercept variances indicate heterogeneity between countries and between institutions. VIF < 5 indicates acceptable collinearity. Exploratory analysis, p–values not adjusted for multiple testing. *p < 0.05.

Entering age, ICU size or clinical position as categorical variables instead of linear scores did not improve model fit (likelihood–ratio tests, all p≥ 0.051)

Abbreviations: ICU, intensive care unit; β, regression coefficient; CI, confidence interval; VIF, variance inflation factor; SD, standard deviation.

Table S5. Concordance of Reported Practice with International Guideline Recommendations

	ESICM 2023	ATS 2017
thresholds for initiation	$PaO_2/FiO_2 \leq 150$ mmHg	$PaO_2/FiO_2 \leq 100$ mmHg
duration	≥ 16 hours	> 12 hours
both	177 (39.7)	65 (14.6)
threshold	247 (55.4)	120 (26.9)
duration	282 (63.2)	296 (66.4)

Values are in n (%) of 446 respondents using prone positioning. Recommendation regarding prone positioning in the ESICM 2023 [20] and the ATS 2017 guideline [18]: the PaO_2/FiO_2 thresholds for initiation, and minimum duration of prone positioning. Respondents who specified another initiation threshold were classified as not consistent

Abbreviations: ATS, American Thoracic Society; ESICM, European Society of Intensive Care Medicine; FiO_2, fraction of inspired oxygen; PaO_2, partial pressure of arterial oxygen

Table S6. Concordance with Recommendations in the ESICM Guideline and Use of Awake Prone Positioning

	concordant with ESICM 2023 guideline 177/446 (39.7)	use of awake prone positioning 297/446 (66.6)
Country	$p < 0.001$	$p = 0.014$
Italy	36/128 (28.1)	77/128 (60.2)
Germany	48/83 (57.8)	67/83 (80.7)
Austria	25/52 (48.1)	39/52 (75.0)
Norway	15/32 (46.9)	22/32 (68.8)
Czech Republic	4/27 (14.8)	18/27 (66.7)
other	49/124 (39.5)	74/124 (59.7)
Number of ICU beds	$p = 0.067$	$p = 0.426$
< 10	39/106 (36.8)	74/106 (69.8)
10–20	46/138 (33.3)	90/138 (65.2)
21–30	23/63 (36.5)	39/63 (61.9)
31–40	15/29 (51.7)	16/29 (55.2)
> 40	54/110 (49.1)	78/110 (70.9)
Hospital type	$p = 0.927$	$p = 0.378$
academic	105/267 (39.3)	173/267 (64.8)
non–academic	72/179 (40.2)	124/179 (69.3)
ECMO availability	$p = 0.281$	$p = 0.948$
yes	89/209 (42.6)	140/209 (67.0)
no	88/237 (37.1)	157/237 (66.2)
*Number of healthcare workers**	$p = 0.679$	$p = 0.384$
≤ 4	82/200 (41.0)	138/200 (69.0)
≥ 5	95/246 (38.6)	159/246 (64.6)

P–values are from the χ^2 test. ESICM 2023 [20]; *, number of healthcare workers reported to be involved for turning a patient.

Abbreviations: ICU, intensive care unit; ECMO, extracorporeal membrane oxygenation.

Table S7. Session duration: linear model and ordinal sensitivity model (N = 425)

	β, hours (95% CI)	p	OR (95% CI)	p
	linear mixed model (Table S4)		ordinal sensitivity model	
ICU size	0.198 (−0.079 to 0.475)	0.162	1.072 (0.938–1.226)	0.308
Age	−0.291 (−0.511 to −0.071)	0.010	0.865 (0.774–0.967)	0.011
Clinical position	0.560 (0.018 to 1.103)	0.044	1.314 (1.000–1.727)	0.050

Both models include country and institution nested within country as random intercepts. Ordinal sensitivity model: duration grouped as < 12 h (n = 72), 12–15 h (n = 88), 16 h (n = 200) and > 16 h (n = 65); OR > 1 indicates higher odds of a longer session. Residuals of the linear model: skewness −0.20, kurtosis 4.00 (normal distribution: 0 and 3). Exploratory analysis; p–values not adjusted for multiple testing. Abbreviations: CI, confidence interval; ICU, intensive care unit; OR, odds ratio; β, regression coefficient.
